# Accuracy of nursing diagnoses of hospitalized people with HIV: implications for care

**DOI:** 10.1590/0034-7167-2025-0133

**Published:** 2025-12-08

**Authors:** Bruna Lixinski Zuge, Jarbas da Silva Ziani, Jenifer Harter, Maria Helena Gehlen, Talita Portela Cassola, Claudia Zamberlan

**Affiliations:** I Universidade Franciscana. Santa Maria, Rio Grande do Sul, Brazil; II Universidade Federal de Santa Maria. Santa Maria, Rio Grande do Sul, Brazil; III Universidade Federal do Pampa. Uruguaiana, Rio Grande do Sul, Brazil

**Keywords:** Nursing Diagnosis, HIV, Acquired Immunodeficiency Syndrome, Hospitalization, Nursing., Diagnóstico de Enfermería, VIH, Síndrome de Inmunodeficiencia Adquirida, Hospitalización, Enfermería.

## Abstract

**Objectives:**

to assess the accuracy of nursing diagnoses listed for the care of people living with HIV during hospitalization.

**Methods:**

a cross-sectional, documentary, retrospective research conducted in a hospital in Rio Grande do Sul, Brazil. Data were collected from medical records of hospitalized individuals living with HIV. The Nursing Diagnostic Accuracy Scale and the NANDA-I taxonomy were used.

**Results:**

the sample consisted of 101 medical records and 218 diagnoses assessed. The prevalent diagnostic hypothesis as a precursor to the need for hospitalization was immunosuppression. The prevalent nursing diagnoses were “Risk for infection” (15.60%), “Risk for impaired skin integrity” (17.80%), and “Acute pain” (5.96%), with a predominance of accurate nursing diagnoses (60.55%).

**Conclusions:**

most diagnoses were accurate, while the null accuracy category was relevant in the sample, suggesting that knowledge on this topic needs to be strengthened in professional practice.

## RESUMEN

**Objetivos:**

evaluar la precisión de los diagnósticos de enfermería para la atención de personas con VIH durante la hospitalización.

**Métodos:**

investigación transversal, documental y retrospectiva realizada en un hospital de Rio Grande do Sul, Brasil. Se recopilaron datos de las historias clínicas de personas con VIH hospitalizadas. Se utilizaron la Escala de Precisión Diagnóstica de Enfermería y la taxonomía NANDA-I.

**Resultados:**

la muestra consistió en 101 historias clínicas y se evaluaron 218 diagnósticos. La hipótesis diagnóstica predominante, precursora de la necesidad de hospitalización, fue la inmunosupresión. Los diagnósticos de enfermería prevalentes fueron “Riesgo de infección” (15,60%), “Riesgo de deterioro de la integridad cutánea” (17,80%) y “Dolor agudo” (5,96%), con un predominio de diagnósticos de enfermería precisos (60,55%).

**Conclusiones:**

la mayoría de los diagnósticos fueron precisos, mientras que la categoría de precisión cero fue relevante en la muestra, lo que sugiere que es necesario fortalecer los conocimientos sobre este tema en la práctica profesional.

## INTRODUCTION

Human Immunodeficiency Virus (HIV) infection has undergone an epidemiological transition. During this transition, it has gone from being an acute disease, with inevitable and progressive progression to Acquired Immunodeficiency Syndrome (AIDS), to a manageable chronic health condition, thanks to advances in prevention and treatment^(1)^. However, the infection continues to pose a global public health concern^(2)^. Global statistics show that by 2023 there were approximately 39.9 million people living with HIV^(1)^.

Although a reduction of up to 60% in new HIV infections has been observed compared to 1995, the incidence of the disease remains high. In Brazil, in 2022, 43,403 new cases of infection were reported, with 10,994 deaths from AIDS-related causes^(3)^. A national study showed that, between 2016 and 2020, 144,671 hospitalizations were recorded due to HIV in Brazil, with emphasis on the South and North regions, which had the highest mortality rates in the country^(4)^.

In this context, it is considered that the chronic nature of HIV, its complexity, the challenges involved in approaching the infection and the possible structural weaknesses of healthcare services can culminate in clinical complications and the need for hospitalizations for people living with HIV^(5)^. In hospital settings, nursing care is highlighted. Resolution 736/2024 of the Federal Nursing Council states that the Nursing Process (NP) must be carried out deliberately and systematically in every socio-environmental context in which nursing care occurs^(6)^.

Nursing diagnoses (NDs) represent the second stage of the NP and are an activity exclusive to nurses. They involve clinical assessment of individuals’, families’, and communities’ health and nursing care needs^(7)^. NDs should consider existing problems, vulnerabilities, and potential areas for improvement. Therefore, it is important to emphasize that identifying NDs should be a daily practice for nurses, as they guide decision-making and, consequently, assertiveness in nursing actions^(7)^.

In addition to the use of NDs, the importance of their accuracy is highlighted. The accuracy of an ND is defined as an evaluator’s judgment regarding the degree of relevance, specificity, and consistency of existing clues for defining each diagnosis. In other words, a diagnosis is considered accurate when it accurately reflects patients’ clinical condition^(8)^. Therefore, the accuracy indicator is essential, given its potential to aid nurses’ NP and demonstrate whether professionals’ diagnostic reasoning is effective^(9)^.

Considering the above, this research is justified by the importance of diagnostic accuracy. Although the literature includes some studies in this field, recent research has addressed the topic through the analysis of a specific ND^(10)^ or focusing on the accuracy of the defining characteristics of a given ND^(10,11)^, as well as in different health contexts^(7,9)^ than those studied here. The literature review reinforces the importance of new and ongoing studies on the topic, seeking to support nursing professionals regarding the best interventions and outcomes for people living with HIV^(12)^.

Furthermore, assessing ND accuracy in different clinical practice contexts can strengthen and solidify knowledge, promoting qualified nursing practice^(7)^. Finally, it is noteworthy that this research is included in target 3.3 of the Sustainable Development Goals, which indicates that ending AIDS as a public health threat by 2030 is possible, and, to this end, it emphasizes respect for science, data, and scientific evidence^(13)^.

## OBJECTIVES

To assess the accuracy of the NDs listed for the care of people living with HIV during hospitalization.

## METHODS

### Ethical aspects

The research was conducted in accordance with Resolution 466/2012^(14)^, following the ethical guidelines governing research involving human beings. The project and conduct were approved by the *Universidade Franciscana* Research Ethics Committee, the authors’ educational institution. Furthermore, it was approved by the teaching coordination of the hospital where it was conducted. The use of the Informed Consent Form was waived, as this was a secondary data collection study from electronic medical records, in which people living with HIV were not identified or contacted. However, the authors agreed to sign the Term Sheet.

### Study design, period and location

This is a cross-sectional, documentary, and retrospective study. STrengthening the Reporting of OBservational studies in Epidemiology^(15)^ recommendations were followed. The study was conducted in a hospital that, in addition to providing healthcare, also serves as a teaching hospital, affiliated with a university that trains professionals in various health fields. The institution provides all of its services within the Brazilian Health System and is located in a municipality in the central region of the state of Rio Grande do Sul.

The hospitalization period established for data collection and analysis was from January 1, 2022, to January 31, 2023. This time frame was established because, in 2020 and 2021, the unit became a reference for hospitalizations of patients with COVID-19, considering that the pandemic context could interfere with data of interest related to HIV.

To select eligible medical records, a manual analysis of all electronic medical records of patients admitted during the period was performed, seeking to select records of people living with HIV. To minimize potential selection bias, two researchers performed this step independently. Data collection took place from February to June 2023.

### Population and selection criteria

The research data were collected from electronic medical records of people living with HIV who were hospitalized in the clinical inpatient unit of the aforementioned hospital. It should be noted that the reason for the hospitalization analyzed did not necessarily need to be due to HIV. Inclusion criteria comprised medical records of people living with HIV aged 18 or older, of both sexes, and who had NDs listed at the time of their admission. Exclusion criterion was defined when an ND was not listed in NANDA-International (NANDA-I) 2018-2020^(16)^. The use of this version is justified, even if the last publication includes the 2024-2026 edition^(17)^, since the system provided by the hospital uses the first version mentioned above.

### Study protocol

Information was collected on sociodemographic variables, such as sex (male/female), age, skin color (white/black/brown/other), education (less than eight years of schooling/more than eight years), and reason for hospitalization/diagnostic hypothesis. If more than one hypothesis was considered, the diagnosis most associated with the main complaint was considered.

To assess diagnostic accuracy, the Nursing Diagnostic Accuracy Scale, Version 2 (In Portuguese, *Escala de Acurácia de Diagnóstico de Enfermagem, Versão 2* - EADE-2)^(8)^ was used. It is important to note that the author authorized the use of the scale by contacting us via email. The EADE-2 was developed nationally by experts in 2006 and validated in a previous study^(18)^ that same year. In 2010, it was improved, resulting in version 2, which was used in this study.

The scale can be used with any ND classification, assessing the degree to which an ND is supported by patients’ clinical data set. To determine diagnostic accuracy, each ND listed in medical records was individually assessed for: 1. Clue presence: patient manifestation represented by signs, traces, or symptoms of the ND being assessed; 2. Clue relevance: degree to which the clues are necessary to indicate the ND; 3. Clue specificity: degree to which the clues are characteristic of the ND; and 4. Clue coherence: degree to which the clues are consistent with the ND and the data set. Subsequently, items were added up, with each score indicating a degree of accuracy, subsequently indicating an accuracy category^(8)^.

The scale presents nine possible degrees and four accuracy categories: null (0); low (1); moderate (2, 4.5 and 5.5); and high (9, 10, 12.5 and 13.5). The scale also allows researchers to reflect on whether or not they would maintain the listed NDs; however, this item does not score^(8)^. Assessments were conducted by reviewing multidisciplinary assessments (medical, nursing, psychology, and social work) conducted during patients’ hospitalization. In addition to signs and symptoms reported in the assessments, the presence of diseases, infections, and associated conditions, the reason for hospitalization, social vulnerabilities, laboratory test results, and history and physical examination were also considered.

It is noteworthy that the assessment of each ND was carried out concomitantly by two nurses, authors of this study, through discussions and careful analysis, considering the clues from medical records, in association with concepts, taxonomies, defining characteristics, and related factors according to NANDA-I 2018-2020^(16)^.

### Analysis of results and statistics

The generated data were entered into Microsoft Office Excel^®^ spreadsheets for tabulation and organization and later transferred to GraphPad Prism (GraphPad Software, San Diego, USA). The analysis was performed using descriptive statistics, using relative and absolute frequencies, as well as mean and standard deviation measures.

## RESULTS

During the study period, a population of 1,328 patients was admitted to the clinical unit; of these, 102 medical records of people living with HIV were analyzed, one of which was excluded because it did not contain an ND listed at the time of admission. Concerning the NDs, 222 were screened, and of these, four were excluded because they were not NDs (“Prevent falls” (1) and “Imbalanced nutritional status” (3)). Thus, the study sample consisted of 101 medical records and 218 NDs.

Of the 101 patients in the sample, the majority were male, white, with more than eight years of education, aged between 18 and 78 years, with a mean age of 43.14 and a standard deviation of 12.77. Immunosuppression was the prevalent condition that led to the need for hospitalization, as shown in Table 1.

**Table 1:** Sociodemographic variables followed by reasons/diagnostic hypothesis for hospital admissions, Santa Maria, Rio Grande do Sul, Brazil, 2025

Sociodemographic variables	n	%	Reason for admission/diagnostic hypothesis	n	%
**Sex**			Immunosuppression	30	29,70%
Male	59	58.41%	Pneumonia	10	9.90%
Female	42	41.59%	Tuberculosis	6	5.94%
**Race/color**			Opportunistic infection under investigation	4	3.96%
White	55	54.45%	Cryptococcosis	3	2.97%
Black	21	20.79%	Malnutrition	3	2.97%
Brown	25	24.75%	Hepatitis	3	2.97%
**Education**			Herpes-zoster	3	2.97%
Less than eight years	41	40.59%	Pancreatitis	3	2.97%
More than eight years	50	59.41%	Kaposi’s sarcoma	3	2.97%
**Age group**			Sexual abuse	2	1.98%
18-27	12	11.88%	Oral and esophageal candidiasis	2	1.98%
28-37	18	17.82%	Neurotoxoplasmosis	2	1.98%
38-47	34	33.66%	Pneumocystis	2	1.98%
48-57	21	20.79%	Sepsis	2	1.98%
>58	16	15.84%	Others	23	22.77%

A total of 34 NDs were identified. Of these, 15.13% were problem-focused (signs/symptoms, diagnostic hypothesis, and social vulnerabilities), while 5.04% corresponded to risks. NDs focused on health promotion and syndromes were less frequent, with only one ND recorded in each category.

However, it is worth noting that, although risk diagnoses represented 5.04% of all NDs analyzed, when disaggregated, they are more prevalent, represented by “Risk for infection” (15.60%) and “Risk for impaired skin integrity” (7.80%), followed by “Acute pain” (5.96%), which represents a ND focused on the problem (Table 2).

**Table 2:** Nursing diagnosis titles listed (N=218) according to type, their respective frequencies, degrees and accuracy categories, Santa Maria, Rio Grande do Sul, Brazil, 2025

ND titles			Accuracy degree		Accuracy categories			
	n	%	Mean	Standard deviation	Null	Low	Moderate	High
Problem-focused ND								
Acute pain	13	5.96%	10.38	5.92	3	-	-	10
Impaired skin integrity	10	4.59%	10.80	5.69	2	-	-	8
Ineffective protection	9	4.13%	7.94	6.62	2	-	2	5
Ineffective breathing pattern	8	3.67%	8.12	6.56	2	1	-	5
Ineffective sexuality pattern	7	3.21%	2.71	5.17	5	-	1	1
Defensive coping	6	2.75%	2.25	5.51	5	-	-	1
Impaired gas exchange	6	2.75%	5.58	6.46	2	1	1	2
Hopelessness	5	2.29%	10.8	6.03	1	-	-	4
Impaired spontaneous ventilation	5	2.29%	12.8	1.56	-	-	-	5
Ineffective coping	4	1.83%	5.87	6.93	2	-	-	2
Ineffective health management	3	1.38%	9	7.79	1	-	-	2
Bathing self-care deficit	3	1.38%	13.5	0	-	-	-	3
Diarrhea	3	1.38%	13.5	0	-	-	-	3
Impaired social interaction	3	1.38%	4.50	7.79	2	-	-	1
Sleep deprivation	3	1.38%	4.5	7.79	2	-	-	1
Situational low self-esteem	2	0.92%	7.75	8.13	-	-	1	1
Risk-prone health behavior	2	0.92%	9.5	5.65	-	-	1	1
Acute confusion	2	0.92%	0	0	2	-	-	-
Ineffective management of therapeutic regimen	2	0.92%	13.5	0	-	-	-	2
Impaired swallowing	2	0.92%	9.50	5.65	-	-	1	1
**Problem-focused ND**								
Impaired physical mobility	2	0.92%	13.5	0	-	-	-	2
Imbalanced nutrition: less than body requirements	2	0.92%	6.75	9.54	1	-	-	1
Urinary retention	2	0.92%	6.75	9.54	1	-	-	1
Spiritual distress	2	0.92%	6.75	9.54	1	-	-	1
Constipation	1	0.46%	13.5	0	-	-	-	1
Impaired walking	1	0.46%	13.5	0	-	-	-	1
Inadequate secondary defenses	1	0.46%	13.5	0	-	-	-	1
Impaired dentition	1	0.46%	13.5	0	-	-	-	1
Impaired oral mucous membrane integrity	1	0.46%	0	0	1	-	-	-
Ineffective health maintenance	1	0.46%	13.5	0	-	-	-	1
Impaired tissue integrity	1	0.46%	13.5	0	-	-	-	1
Activity intolerance	1	0.46%	10	0	-	-	-	1
**Risk ND**								
Risk for infection	34	15.60%	8.16	5.81	6	2	5	21
Risk for impaired skin integrity	17	7.80%	4.97	6.14	7	1	3	6
Risk for falls	12	5.50%	8.91	6.59	3	-	-	9
Risk for loneliness	12	5.50%	9.00	6.64	4	-	-	8
Risk for compromised human dignity	5	2.29%	8.5	6.88	1	-	1	3
Risk for powerlessness	5	2.29%	8.10	7.39	2	-	-	3
Risk for aspiration	3	1.38%	7.83	7.00	1	-	-	2
Risk for suicide	2	0.92%	13.5	0	-	-	-	2
Risk for ineffective cerebral tissue perfusion	2	0.92%	6.75	9.54	1	-	-	1
Risk for imbalanced body temperature	1	0.46%	13.5	0	-	-	-	1
Risk for shock	1	0.46%	13.5	0	-	-	-	1
**Health promotion ND**								
Readiness for enhanced coping	5	2.29%	0	0	5	-	-	-
**Syndrome ND**								
Rape trauma syndrome	3	1.38%	13.5	0	-	-	-	3

*ND - nursing diagnosis.*

The NANDA-I edition, version 2018-2020^(16)^, has 13 domains, 12 of which were covered by the NDs analyzed. The only domain for which no NDs were listed was “Growth/Development”.

High-accuracy NDs predominated, representing 60% of all categories, followed by the null-accuracy category (29.82%) (Table 3). The authors also used the EADE-2 column, which asked whether to maintain each ND. Thus, more than 58% of the NDs listed by nurses were maintained by the researchers.

**Table 3:** Frequency of accuracy degrees and categories according to the nursing diagnoses assessed (N = 218), Santa Maria, Rio Grande do Sul, Brazil, 2025

Accuracy		Frequency	
**Category**	**Degree**	**n**	**%**
Null	00	65	29.82%
Low	01	05	2.30%
Moderate	02	11	5.04%
	4.5	0	0
	5.5	5	2.30%
High	09	3	1.37%
	10	8	3.66%
	12.5	2	0.91%
	13.5	119	54.60%

## DISCUSSION

Among the sociodemographic characteristics of the 101 medical records analyzed, the sample showed a predominance of males, supporting what has been demonstrated in the literature over time. A Brazilian study that analyzed AIDS cases in the country between 2015 and 2020 showed a prevalence of 71% of cases in males^(19)^. The same continued to be observed from 2020 to 2023, configuring a significant male predominance of HIV and AIDS cases in the country^(20)^.

A recent study demonstrated that, while male people living with HIV were highly associated with the risk of urgent hospitalization and death, white skin color was identified as a protective factor, as it presented four times less chance of death compared to black/mixed skin color^(21)^. White skin people represented a representative portion of this study, making up more than 50% of the sample.

The predominant age groups were those between 38 and 47 years old, followed by those between 48 and 57 years old. This result is in line with Brazilian data, in which the 40 to 49 age group represented the most affected by AIDS cases in all regions of the state, a fact that possibly suggests risky behaviors and greater exposure to the virus throughout life^(20)^. Meanwhile, conversely, younger people (20-24 years old) and older people (60 years old or older) tend to represent smaller proportions of cases, which may suggest that young people are less exposed due to awareness of prevention methods, in addition to a lower incidence of infection among older adults^(20,21)^.

When analyzing the diagnostic hypothesis associated with the need for hospitalization, it was observed that the main causes of hospitalization in Brazil and in international settings are convergent. A study from Rio Grande do Norte^(22)^ found neurotoxoplasmosis and pneumonia as the main causes of hospitalization, while a Chinese study detected pneumocystis pneumonia, tuberculosis, and cytomegalovirus infection^(23)^, similar to the patient sample in this study, which had immunosuppression, pneumonia, and tuberculosis as the prevalent precursor causes of the need for hospitalization.

A higher proportion of hospitalizations was observed in immunocompromised patients without prior treatment with antiretroviral therapy (ART), indicating that treatment can be considered a protective factor against hospitalizations^(23)^. A national study demonstrated that indicators such as high viral load and CD4+ counts below 200 copies/mm^3^ are predictors of the need for urgent hospitalization. The study also found that urgent hospital admission, having an HIV diagnosis discovered during hospitalization, and not using ART are directly associated with in-hospital death^(21)^.

It is also worth pointing out factors such as greater distance between residence and healthcare service, first hospital admission, COVID-19, cardiovascular, renal, and hepatic diseases, anemia, co-infections, malnutrition, diabetes mellitus, and neurological disorders, which were significantly associated with hospital mortality among people living with HIV^(24)^.

Concerning nursing, research led by nurses emphasized that a closer relationship with patients can positively change the experience of individuals regarding the early initiation of treatment with ART^(25)^, making it essential that this professional’s work involves the entire care cycle^(26)^. In view of this, it has been emphasized that, for healthcare continuity after hospital discharge, effective referral to the services that, within the Health Care Network (In Portuguese, *Rede de Atenção à Saúde* - RAS), make up HIV care is essential, constituting a primary strategy to reduce new complications in this population^(27)^.

In relation to the NDs analyzed, it was demonstrated that the NDs listed by the nurses were accurate, expressed by the predominance of the high accuracy category, indicating nurses’ refined reasoning. However, it was observed that the null accuracy category represented the second most common category, suggesting that the information in some medical records did not contain clues that indicated the ND listed, making any assessment in the EADE-2 impossible. It is indicated that, for highly accurate NDs, the documented and assessed clues must have high relevance, specificity, and coherence^(7)^.

Several difficulties inherent to NDs are mentioned, with important obstacles being described, such as lack of time due to excessive activities, high number of patients, reduced staff, and little knowledge and experience of the team due to the use of NDs^(28)^. Research found that some nurses had a negative perception of NDs, as they had doubts as to whether the diagnoses actually helped in understanding patients’ conditions^(29)^.

The risk diagnosis category represented the NDs most frequently listed by nurses, with “Risk for infection” and “Risk for impaired skin integrity” being the most frequently used, respectively. “Risk for infection” was the most frequently used ND, as immunosuppression was the prevalent cause of hospitalization in this population. Individuals with HIV are at higher risk of developing opportunistic infections, contributing to increased hospital stays and mortality^(22,28)^. In this context, accurate NDs based on the correct interpretation of human responses are essential, since accurate diagnoses contribute to better prognoses^(30)^.

Problem-focused NDs represented the second most frequently used category, with “Acute pain” and “Impaired skin integrity” being the most frequently listed. It is worth noting that changes in skin integrity are commonly associated with hospitalized patients due to the invasive procedures inherent in the environment. This situation requires attention, as invasive procedures can lead to the development of healthcare-associated infections, especially in people living with HIV due to immunosuppression^(22)^. These data indicate that one in four people living with HIV is at risk of death as an outcome during hospitalization^(24)^.

A comprehensive literature review on NDs in different patient populations and healthcare settings found that “Acute pain” was the only ND identified in all studies analyzed^(31)^, contrasting with the results of this study, in which the aforementioned ND was listed in only 13 medical records. Nurses’ interventions in pain management tend to prioritize assessment and pharmacological treatment, suggesting the need for team training with an emphasis on non-pharmacological pain management^(32)^. In the context of HIV care, chronic pain is recognized as a common comorbidity and a threat to the quality of life of these people^(33)^.

Biological, psychological, and social factors are thought to be associated with increased pain, such as HIV-related stigma, lack of social support, reduced access to services, dysregulated immune activity, inflammation, and tissue damage caused by HIV-related co-infections^(33)^. Since pain is a subjective experience and its perception is considered complex and multidimensional, understanding these influences is essential for implementing personalized and effective strategies for pain control and management^(34)^.

It was also identified that NDs related to health promotion and syndrome represented the minority of the sample, with only one diagnosis of each type listed, respectively represented by “Readiness for enhanced coping” and “Rape trauma syndrome”. Similarly, among the diagnoses listed for the care of patients in the Intensive Care Unit, the absence of NDs focused on health promotion stood out, with a predominance of risk diagnoses and problem-focused diagnoses^(7)^. This may indicate that nurses are focusing attention only on the complications presented, not considering the possible ways of promoting health, these NDs being also relevant in the context of avoiding/minimizing other health problems.

Brazil has dedicated efforts to research on the accuracy of NDs, reinforcing the importance of the quality of this practice for Brazilian nursing^(35)^. In summary, accurate NDs contribute to the development of a care plan targeted at people living with HIV, fostering the management and care organization of nurses’ actions, by directing professionals to a set of phenomena to which their attention needs to be attentive, considering the entire context of these people^(35)^.

### Study limitations

Since this is a study that collected data from medical records, some pertinent information to reinforce the study data was missing and/or its accuracy is uncertain; therefore, it was not presented in this manuscript, such as marital status, sexual orientation, occupational status, and adherence to ART. Furthermore, the assessment of risk factors and defining characteristics considered for each ND could complement accuracy assessment.

Caution is advised when generalizing the results, as the study did not include inferential statistical analysis, which may have limited the depth of the findings. It should be noted, however, that the results obtained are consistent with the literature.

### Contributions to nursing

This study contributes to the advancement of nursing and NANDA-I taxonomy by providing evidence that can improve the quality of nursing care practice as a science in the national setting. The study emphasizes and reinforces the importance of nursing professionals’ understanding of this topic, as it constitutes a guiding tool for care, promoting comfort, well-being, and quality of life during hospitalization and, especially, in the daily lives of people living with HIV.

## CONCLUSIONS

The NDs analyzed were mostly accurate, indicating that nurses possess refined clinical reasoning. However, the null accuracy category was significant in the sample, suggesting that knowledge on the topic needs to be strengthened in professional practice, with the importance of assertive NDs recognized by managers. It is also noteworthy that the topic should be included in undergraduate and graduate nursing programs, extending to professional practice through continuing education in health, due to its relevance to nursing practice.

The prevalent NDs listed by nurses were “Risk for infection”, “Risk for impaired skin integrity”, and “Acute pain”, focusing on risks and problems, suggesting that nursing care is also geared toward these issues. This fosters the need for health promotion, including in hospital settings, considering that, due to the chronic nature of HIV infection, it is crucial that, after discharge, these individuals continue to seek other services within RAS.

The information obtained in this study may also shed light on the profile of the population living with HIV who is hospitalized due to worsening infection, guiding researchers and policymakers regarding this population’s health needs.

For future research, we suggest that the authors consider studies that address the characteristics and risk factors commonly presented by people living with HIV, seeking to meet their health needs through targeted and effective nursing interventions.

## Data Availability

The research data are available within the article.
