# Supplementary material for: Accuracy of nursing diagnoses of hospitalized people with HIV: implications for care
Source: Rev Bras Enferm. 2025 Dec 8;78(Suppl 3):e20250133. doi: 10.1590/0034-7167-2025-0133 (PMC12707848; doi:10.1590/0034-7167-2025-0133)
Supplement: Translation

# Translation: Acurácia dos diagnósticos de enfermagem de pessoas hospitalizadas com HIV: implicações para a assistência

**DOI:** 10.1590/0034-7167-2025-0133pt

**Authors:** Bruna Lixinski Zuge, Jarbas da Silva Ziani, Jenifer Harter, Maria Helena Gehlen, Talita Portela Cassola, Claudia Zamberlan

**Affiliations:** I Universidade Franciscana. Santa Maria, Rio Grande do Sul, Brasil; II Universidade Federal de Santa Maria. Santa Maria, Rio Grande do Sul, Brasil; III Universidade Federal do Pampa. Uruguaiana, Rio Grande do Sul, Brasil

**Keywords:** Diagnóstico de Enfermagem, HIV, Síndrome de Imunodeficiência Adquirida, Hospitalização, Enfermagem.

## Abstract

**Objetivos:**

avaliar a acurácia dos diagnósticos de enfermagem elencados para o cuidado de pessoas vivendo com HIV durante a internação hospitalar.

**Métodos:**

pesquisa transversal, documental e retrospectiva, desenvolvida em uma instituição hospitalar situada no Rio Grande do Sul, Brasil. Foram coletados dados de prontuários de pessoas vivendo com HIV hospitalizadas. Utilizaram-se a Escala de Acurácia de Diagnóstico de Enfermagem e a taxonomia NANDA-I.

**Resultados:**

a amostra foi composta por 101 prontuários e 218 diagnósticos avaliados. A hipótese diagnóstica prevalente como precursora de necessidade de internação foi a imunossupressão. Os diagnósticos de enfermagem prevalentes foram “Risco de infeção” (15,60%), “Risco de integridade da pele prejudicada” (17,80%) e “Dor aguda” (5,96%), com predomínio de diagnósticos de enfermagem acurados (60,55%).

**Conclusões:**

os diagnósticos foram, em sua maioria, acurados, ao passo que a categoria de acurácia nula apresentou relevância na amostra, sugerindo que o conhecimento acerca do tema precisa ser fortalecido na prática profissional.

## INTRODUÇÃO

A infecção pelo Vírus da Imunodeficiência Humana (HIV) passou por uma transição epidemiológica. Nessa transição, ela deixou de ser uma doença aguda, com avanço inevitável e progressivo para o estágio de Síndrome da Imunodeficiência Adquirida (AIDS), para se tornar uma condição de saúde crônica gerenciável, devido aos avanços nas formas de prevenção e tratamento^(1)^. No entanto, a infecção continua se apresentando como um problema para a saúde pública mundial^(2)^. Estatísticas globais demonstram que, até 2023, havia cerca de 39,9 milhões de pessoas vivendo com HIV^(1)^.

Embora tenha sido observada uma redução de até 60% nas novas infecções por HIV em comparação com 1995, a incidência da doença permanece elevada. No Brasil, em 2022, foram notificados 43.403 novos casos de infecção, com 10.994 mortes por causas relacionadas à Aids^(3)^. Estudo nacional demonstrou que, entre 2016 e 2020, 144.671 hospitalizações foram registradas em decorrência do HIV no Brasil, com destaque para as regiões Sul e Norte, que apresentaram os maiores coeficientes de mortalidade do país^(4)^.

Nesse âmbito, considera-se que o caráter crônico do HIV, a sua complexidade, os desafios que envolvem a abordagem da infecção e as possíveis fragilidades estruturais dos serviços de saúde podem culminar em complicações clínicas e na necessidade de internações hospitalares às pessoas que vivem com HIV^(5)^. No contexto hospitalar, os cuidados de enfermagem ganham destaque. A Resolução n^o^ 736/2024 do Conselho Federal de Enfermagem aponta que o Processo de Enfermagem (PE) deve ser realizado de modo deliberado e sistemático em todo contexto socioambiental em que ocorre o cuidado de enfermagem^(6)^.

Os diagnósticos de enfermagem (DEs) representam a segunda etapa do PE, constituindo-se uma atividade privativa dos enfermeiros. Envolve a avaliação clínica das necessidades de cuidado em saúde e enfermagem da pessoa, famílias ou coletividades^(7)^. Os DEs devem considerar os problemas existentes, as vulnerabilidades, bem como as condições de melhora. Dessa forma, ressalta-se que a identificação dos DEs deve ser uma prática diária no cotidiano dos enfermeiros, visto que orientam a tomada de decisão e, consequentemente, a assertividade nas ações de enfermagem^(7)^.

Além da utilização dos DEs, destaca-se a importância de sua acurácia. A acurácia de um DE é definida como o julgamento de um avaliador quanto ao grau de relevância, especificidade e consistência das pistas existentes para definição de cada diagnóstico. Ou seja, um diagnóstico é considerado acurado quando reflete com precisão a condição clínica do paciente^(8)^. Desse modo, é fundamental o indicador de acurácia, visto seu potencial em auxiliar no PE dos enfermeiros e evidenciar se o raciocínio diagnóstico dos profissionais está sendo efetivo^(9)^.

Considerando o exposto, esta pesquisa se justifica devido à importância da acurácia diagnóstica. Embora a literatura contemple alguns estudos no âmbito, pesquisas recentes abordaram a temática por meio da análise de um DE específico^(10)^ ou com foco na acurácia das características definidoras de determinado DE^(10,11)^, além de contextos de saúde diferentes^(7,9)^ do aqui pesquisado. Revisão de literatura reforça a importância de novos e constantes estudos sobre o tema, buscando subsidiar os profissionais de enfermagem quanto às melhores intervenções e resultados para pessoas vivendo com HIV^(12)^.

Além disso, a avaliação da acurácia dos DEs em diferentes contextos da prática clínica pode fortalecer e solidificar os conhecimentos, promovendo uma prática de enfermagem qualificada^(7)^. Por fim, ressalta-se que esta pesquisa está contemplada na meta 3.3 dos Objetivos de Desenvolvimento Sustentável, que indica que acabar com a aids como ameaça à saúde pública até 2030 é possível, e, para isso, destaca o respeito à ciência, aos dados e às evidências científicas^(13)^.

## OBJETIVOS

Avaliar a acurácia dos DEs elencados para o cuidado de pessoas vivendo com HIV durante a internação hospitalar.

## MÉTODOS

### Aspectos éticos

A pesquisa foi conduzida em consonância com a Resolução n^o^ 466/2012^(14)^, seguindo as diretrizes de ética que regem pesquisas com seres humanos, sendo o projeto e a condução aprovados pelo Comitê de Ética em Pesquisa da Universidade Franciscana, instituição de ensino de vínculo dos autores. Além disso, foi aprovada pela coordenação de ensino do hospital em que foi realizada. Dispensou-se o uso do Termo de Consentimento Livre e Esclarecido, por tratar-se de coleta de dados secundários em prontuários eletrônicos, em que as pessoas que vivem com HIV não foram identificadas e contatadas. Contudo, os autores se comprometeram a assinar o Termo de Compromisso.

### Desenho, período e local do estudo

Trata-se de pesquisa transversal, documental e retrospectiva. Utilizaram-se as recomendações da ferramenta *STrengthening the Reporting of OBservational studies in Epidemiology* ^(15)^. A pesquisa foi desenvolvida em uma instituição hospitalar que, além de possuir caráter assistencial, também se constitui um hospital de ensino por estar vinculado a uma universidade formadora de profissionais das diferentes áreas da saúde. A instituição realiza a totalidade de seus atendimentos vinculados ao Sistema Único de Saúde, e está localizada em um município da região central do estado do Rio Grande do Sul.

O período de internação estabelecido para coleta de dados e análise foi de 01 de janeiro de 2022 a 31 de janeiro de 2023. Estabeleceu-se esse recorte temporal porque, em 2020 e 2021, a unidade tornou-se referência para internações de pacientes com COVID-19, considerando que o contexto pandêmico poderia interferir nos dados de interesse relacionados ao HIV.

Para seleção dos prontuários elegíveis, realizou-se análise manual de todos os prontuários eletrônicos de pacientes admitidos no período, buscando selecionar prontuários de pessoas que vivem com HIV. No intuito de minimizar possíveis vieses de seleção, dois pesquisadores realizaram essa etapa de forma independente. A coleta de dados ocorreu no período de fevereiro a junho de 2023.

### População e critérios de seleção

Os dados da pesquisa foram coletados de prontuários eletrônicos de pessoas que vivem com HIV que estiveram hospitalizadas na unidade de internação clínica do referido hospital. Ressalta-se que o motivo da internação analisada não precisava, necessariamente, ser em decorrência do HIV. Definiram-se como critérios de inclusão prontuários de pessoas que vivem com HIV com idade igual ou superior a 18 anos, de ambos os sexos e que tiveram DEs elencados no momento de sua admissão. Como critério de exclusão, definiu-se DE que não estivesse elencado no NANDA-Internacional (NANDA-I) 2018-2020^(16)^. Justifica-se o uso dessa versão, mesmo que a última publicação compreenda a edição 2024-2026^(17)^, visto que o sistema disponibilizado pelo hospital utiliza a primeira versão supracitada.

### Protocolo do estudo

Coletaram-se informações de variáveis sociodemográficas, como sexo (masculino/feminino), idade, cor da pele (branca/preta/parda/outra), escolaridade (menos de oito anos de estudo/mais de oito anos) e motivo da internação/hipótese diagnóstica. No caso de ter sido considerada mais de uma hipótese, considerou-se o diagnóstico mais associado à queixa principal apresentada.

Para avaliar a acurácia diagnóstica, utilizou-se a Escala de Acurácia de Diagnóstico de Enfermagem, Versão 2 (EADE-2)^(8)^. Ressalta-se que a autora autorizou a utilização da escala mediante contato via *e-mail*. A EADE-2 foi desenvolvida nacionalmente por especialistas em 2006 e validada em estudo prévio^(18)^ nesse mesmo ano. Em 2010, foi aprimorada, resultando na versão 2, a qual foi utilizada neste estudo.

A escala pode ser utilizada com qualquer classificação de DE, avaliando o grau com que um DE é sustentado pelo conjunto de dados clínicos do paciente. Para determinar a acurácia diagnóstica, cada DE elencado nos prontuários foi avaliado individualmente quanto à: 1. Presença de pista: manifestação do paciente representada por indícios, vestígios ou sinais e sintomas do DE avaliado; 2. Relevância da pista: grau com que as pistas são necessárias para indicar o DE; 3. Especificidade da pista: grau com que as pistas são características do DE; e 4. Coerência da pista: grau com que as pistas são consistentes com o DE e com o conjunto de informações. Na sequência, realizou-se a soma dos itens, em que cada pontuação aponta um grau de acurácia, indicando, na sequência, uma categoria de acurácia^(8)^.

A escala apresenta nove possibilidades de graus e quatro categorias de acurácia, sendo nula (0), baixa (1), moderada (2, 4,5 e 5,5) e alta (9, 10, 12,5 e 13,5). A escala permite, ainda, que os pesquisadores reflitam se manteriam ou não os DEs elencados; no entanto, esse item não pontua^(8)^. As avaliações ocorreram por meio da leitura das evoluções multiprofissionais (médicas, de enfermagem, de psicologia e de assistência social) realizadas durante o período da internação. Além de sinais e sintomas relatados nas evoluções, consideraram-se a presença de doenças, infecções e agravos associados, motivo da internação hospitalar, vulnerabilidades sociais, resultados de exames laboratoriais, bem como anamnese e exame físico.

Ressalta-se que a avaliação de cada DE foi realizada de forma concomitante por dois enfermeiros, autores deste estudo, por meio de discussões e análise criteriosa, considerando as pistas dos prontuários, em associação com os conceitos, taxonomias, características definidoras e fatores relacionados, conforme NANDA-I 2018-2020^(16)^.

### Análise dos resultados e estatística

Os dados gerados foram lançados em planilhas no *Microsoft Office Excel^®^* para tabulação e organização e, posteriormente, transferidos para o *GraphPad Prism* (*GraphPad Software, San Diego, USA*). Realizou-se a análise por meio de estatística descritiva, utilizando frequências relativas e absolutas, além de medidas de média e desvio padrão.

## RESULTADOS

No período do estudo, uma população de 1.328 pacientes foi admitida na unidade clínica; desses, 102 prontuários de pessoas que vivem com HIV foram analisados, sendo um excluído por não constar DE elencado no momento da internação. Quanto aos DEs, 222 foram rastreados e, desses, foram quatro excluídos por não se tratar de DE (“Prevenir quedas” (1) e “Estado nutricional desequilibrado) (3)). Assim, a amostra do estudo foi composta por 101 prontuários e 218 DEs.

Dos 101 pacientes da amostra, a maior parte correspondeu ao sexo masculino, de cor branca, com mais de oito anos de estudo, faixas etárias entre 18 e 78 anos, com média de idade de 43,14 e desvio padrão de 12,77. A imunossupressão foi a condição prevalente como precursora da necessidade de internação hospitalar, conforme exposto na Tabela 1.

**Tabela 1:** Variáveis sociodemográficas seguidas dos motivos/hipótese diagnóstica das internações hospitalares, Santa Maria, Rio Grande do Sul, Brasil, 2025

Variáveis sociodemográficas	n	%	Motivo da internação/ hipótese diagnóstica	n	%
**Sexo**			Imunossupressão	30	29,70%
Masculino	59	58,41%	Pneumonia	10	9,90%
Feminino	42	41,59%	Tuberculose	6	5,94%
**Raça/cor**			Infecção oportunista em investigação	4	3,96%
Branca	55	54,45%	Criptococose	3	2,97%
Preta	21	20,79%	Desnutrição	3	2,97%
Parda	25	24,75%	Hepatites	3	2,97%
**Escolaridade**			Herpes-zóster	3	2,97%
Menos de oito anos	41	40,59%	Pancreatite	3	2,97%
Mais de oito anos	50	59,41%	Sarkoma de Kaposi	3	2,97%
**Faixa etária**			Abuso sexual	2	1,98%
18-27	12	11,88%	Candidíase oral e esofágica	2	1,98%
28-37	18	17,82%	Neurotoxoplasmose	2	1,98%
38-47	34	33,66%	Pneumocistose	2	1,98%
48-57	21	20,79%	Sepse	2	1,98%
>58	16	15,84%	Outros	23	22,77%

Identificou-se um total de 34 DEs. Desses, 15,13% eram focados no problema (sinais/sintomas, hipótese diagnóstica e vulnerabilidades sociais), enquanto 5,04% correspondiam aos riscos. Os DEs voltados à promoção da saúde e às síndromes foram menos frequentes, com apenas um DE registrado em cada categoria.

Contudo, cabe ressaltar que, embora os diagnósticos de risco tenham representado 5,04% de todos os DEs analisados, quando desagregados, encontram-se em maior prevalência, retratado por “Risco de infeção” (15,60%) e “Risco de integridade da pele prejudicada” (7,80%), seguido por “Dor aguda” (5,96%), o qual representa um DE com foco no problema (Tabela 2).

**Tabela 2:** Títulos dos diagnósticos de enfermagem elencados (N= 218) segundo tipo, suas respectivas frequências, graus e categorias de acurácia, Santa Maria, Rio Grande do Sul, Brasil, 2025

Títulos dos DEs			Grau de acurácia		Categorias de acurácia			
	n	%	Média	Desvio padrão	Nula	Baixa	Moderada	Alta
DE com foco no problema								
Dor aguda	13	5,96%	10,38	5,92	3	-	-	10
Integridade da pele prejudicada	10	4,59%	10,80	5,69	2	-	-	8
Proteção ineficaz	9	4,13%	7,94	6,62	2	-	2	5
Padrão respiratório ineficaz	8	3,67%	8,12	6,56	2	1	-	5
Padrão de sexualidade ineficaz	7	3,21%	2,71	5,17	5	-	1	1
Enfrentamento defensivo	6	2,75%	2,25	5,51	5	-	-	1
Troca de gases prejudicada	6	2,75%	5,58	6,46	2	1	1	2
Desesperança	5	2,29%	10,8	6,03	1	-	-	4
Ventilação espontânea prejudicada	5	2,29%	12,8	1,56	-	-	-	5
Enfrentamento ineficaz	4	1,83%	5,87	6,93	2	-	-	2
Controle ineficaz da saúde	3	1,38%	9	7,79	1	-	-	2
Déficit no autocuidado para banho/higiene	3	1,38%	13,5	0	-	-	-	3
Diarreia	3	1,38%	13,5	0	-	-	-	3
Interação social prejudicada	3	1,38%	4,50	7,79	2	-	-	1
Privação de sono	3	1,38%	4,5	7,79	2	-	-	1
Baixo autoestima situacional	2	0,92%	7,75	8,13	-	-	1	1
Comportamento de saúde propenso a risco	2	0,92%	9,5	5,65	-	-	1	1
Confusão aguda	2	0,92%	0	0	2	-	-	-
Controle ineficaz do regime terapêutico	2	0,92%	13,5	0	-	-	-	2
Dificuldade para deglutir	2	0,92%	9,50	5,65	-	-	1	1
**DE com foco no problema**								
Mobilidade física prejudicada	2	0,92%	13,5	0	-	-	-	2
Nutrição desequilibrada: menor do que as necessidades corporais	2	0,92%	6,75	9,54	1	-	-	1
Retenção urinária	2	0,92%	6,75	9,54	1	-	-	1
Sofrimento espiritual	2	0,92%	6,75	9,54	1	-	-	1
Constipação	1	0,46%	13,5	0	-	-	-	1
Deambulação prejudicada	1	0,46%	13,5	0	-	-	-	1
Defesas secundárias inadequadas	1	0,46%	13,5	0	-	-	-	1
Dentição prejudicada	1	0,46%	13,5	0	-	-	-	1
Integridade da membrana mucosa oral prejudicada	1	0,46%	0	0	1	-	-	-
Manutenção ineficaz da saúde	1	0,46%	13,5	0	-	-	-	1
Integridade tissular prejudicada	1	0,46%	13,5	0	-	-	-	1
Intolerância à atividade	1	0,46%	10	0	-	-	-	1
**DE de risco**								
Risco de infeção	34	15,60%	8,16	5,81	6	2	5	21
Risco de integridade da pele prejudicada	17	7,80%	4,97	6,14	7	1	3	6
Risco de quedas	12	5,50%	8,91	6,59	3	-	-	9
Risco de solidão	12	5,50%	9,00	6,64	4	-	-	8
Risco de dignidade humana comprometida	5	2,29%	8,5	6,88	1	-	1	3
Risco de sentimento de impotência	5	2,29%	8,10	7,39	2	-	-	3
Risco de aspiração	3	1,38%	7,83	7,00	1	-	-	2
Risco de suicídio	2	0,92%	13,5	0	-	-	-	2
Risco de perfusão tissular cerebral ineficaz	2	0,92%	6,75	9,54	1	-	-	1
Risco de desequilíbrio da temperatura corporal	1	0,46%	13,5	0	-	-	-	1
Risco de choque	1	0,46%	13,5	0	-	-	-	1
**DE de promoção da saúde**								
Disposição para enfrentamento melhorado	5	2,29%	0	0	5	-	-	-
**DE de síndrome**								
Síndrome do trauma de estupro	3	1,38%	13,5	0	-	-	-	3

*DE - diagnóstico de enfermagem.*

A edição NANDA-I, versão 2018-2020^(16)^, possui 13 domínios, sendo que 12 foram contemplados pelos DEs analisados. O único domínio para o qual não havia DEs elencados foi o “Crescimento/Desenvolvimento”.

Houve predomínio de DE com alta acurácia, representando 60% de todas as categorias, seguido pela categoria de acurácia nula (29,82%) (Tabela 3). Os autores utilizaram, ainda, a coluna da EADE-2 que se tratava de uma reflexão quanto a manter ou não cada DE. Dessa forma, mais de 58% dos DEs elencados pelos enfermeiros foram mantidos pelos pesquisadores.

**Tabela 3:** Frequência dos graus e categorias de acurácia segundo os diagnósticos de enfermagem avaliados (N =218), Santa Maria, Rio Grande do Sul, Brasil, 2025

Acurácia		Frequência	
**Categoria**	**Grau**	**n**	**%**
Nula	00	65	29,82%
Baixa	01	05	2,30%
Moderada	02	11	5,04%
	4,5	0	0
	5,5	5	2,30%
Alta	09	3	1,37%
	10	8	3,66%
	12,5	2	0,91%
	13,5	119	54,60%

## DISCUSSÃO

Entre as características sociodemográficas dos 101 prontuários analisados, a amostra obteve predominância do sexo masculino, corroborando o demonstrado pela literatura ao longo do tempo. Estudo brasileiro que analisou os casos de aids no país entre 2015 e 2020 evidenciou uma prevalência de 71% dos casos no gênero masculino^(19)^. O mesmo seguiu sendo observado no período de 2020 a 2023, configurando uma predominância masculina significativa dos casos de HIV e aids no país^(20)^.

Estudo recente demonstrou que, enquanto pessoas que vivem com HIV do sexo masculino estiveram altamente associadas ao risco de hospitalização urgente e ao óbito, a cor da pele branca foi identificada como fator de proteção, visto que apresentou quatro vezes menos chances de óbito em comparação aos pretos/pardos^(21)^. Pessoas da pele branca representaram parcela representativa deste estudo, compondo mais de 50% da amostra.

As faixas etárias predominantes foram as situadas entre os 38 e 47 anos, seguidas de 48 a 57 anos. Esse resultado foi ao encontro de dados brasileiros, em que a faixa dos 40 a 49 anos representou a mais afetada por casos de aids em todas as regiões do estado, fato que, possivelmente, sugere comportamentos de risco e maior exposição ao vírus ao longo da vida^(20)^. Enquanto isso, inversamente, os mais jovens (20-24 anos) e os mais velhos (60 anos ou mais) tendem a representar menores proporções dos casos, o que pode sugerir que os jovens se expõem menos devido à conscientização sobre formas de prevenção, além de menor incidência da infecção entre os idosos^(20,21)^.

Ao analisar a hipótese diagnóstica associada à necessidade de internação hospitalar, observou-se que as principais causas de internação no Brasil e em cenários internacionais são convergentes. Estudo do Rio Grande do Norte^(22)^ constatou neurotoxoplasmose e pneumonia como as principais causas de internação, enquanto que estudo chinês detectou pneumonia pneumocística, tuberculose e infecção por citomegalovírus^(23)^, similar com a amostra de pacientes deste estudo, a qual teve imunossupressão, pneumonia e tuberculose como as causas prevalentes precursoras de necessidade de internação hospitalar.

Observou-se maior proporção de hospitalizações em pacientes imunodeprimidos e sem tratamento prévio com terapia antirretroviral (TARV), apontando que o tratamento pode ser considerado como fator protetor contra hospitalizações^(23)^. Estudo nacional demonstrou que indicadores como carga viral alta e CD4+ menor que 200 cópias/mm^3^ são preditores de necessidade de internação urgente. O estudo também constatou que a admissão hospitalar de forma urgente, ter o diagnóstico de HIV descoberto durante a internação e a não utilização da TARV estão diretamente associados ao óbito hospitalar^(21)^.

Cabe ainda apontar para fatores como maior distância entre a residência e o serviço de saúde, primeira admissão hospitalar, covid-19, doenças cardiovasculares, renais, hepáticas, anemia, coinfecções, desnutrição, diabetes mellitus e distúrbios neurológicos, os quais foram significativamente associados à mortalidade hospitalar entre pessoas que vivem com HIV^(24)^.

No que tange à enfermagem, pesquisa liderada por enfermeiros enfatizou que um relacionamento mais próximo com os pacientes pode modificar positivamente a experiência dos indivíduos quanto ao início precoce do tratamento com a TARV^(25)^, sendo fundamental que a atuação desse profissional envolva todo o ciclo de cuidado^(26)^. Diante disso, tem-se enfatizado que, para a continuidade dos cuidados de saúde após a alta hospitalar, é imprescindível um encaminhamento efetivo para os serviços que, dentro da Rede de Atenção à Saúde (RAS), compõem a atenção ao HIV, constituindo-se estratégia primordial para reduzir novas complicações nessa população^(27)^.

Quanto aos DEs analisados, demonstrou-se que os DEs elencados pelos enfermeiros foram acurados, expresso pela predominância da categoria de alta acurácia, indicando um apurado raciocínio dos enfermeiros. Todavia, observou-se que a categoria de acurácia nula representou a segunda categoria mais encontrada, sugerindo que as informações de alguns prontuários não apresentavam pistas que indicassem o DE elencado, impossibilitando qualquer avaliação na EADE-2. Indica-se que, para DEs altamente acurados, as pistas documentadas e avaliadas precisam ter alta relevância, especificidade e coerência^(7)^.

Diversas dificuldades inerentes aos DEs são referidas, sendo descritos obstáculos importantes, como tempo escasso diante do excesso de atividades, alto número de pacientes, equipe diminuída, pouco conhecimento e experiência da equipe devido ao uso dos DEs^(28)^. Pesquisa constatou que alguns enfermeiros possuíam uma percepção negativa sobre os DEs, uma vez que apresentavam dúvidas se os diagnósticos de fato auxiliavam na compreensão das condições dos pacientes^(29)^.

A categoria de diagnósticos de risco representou os DEs mais elencados pelos enfermeiros, sendo, respectivamente, “Risco de infecção” e “Risco de integridade da pele prejudicada” os utilizados com maior frequência. “Risco de infecção” ter correspondido ao DE mais utilizado, pode justificar-se devido a imunossupressão ter se apresentado como a causa prevalente de internação nessa população. Indivíduos com HIV apresentam maior risco de desenvolver infecções oportunistas, contribuindo para o aumento do tempo de hospitalização e mortalidade^(22,28)^. Nesse contexto, faz-se imprescindível DEs acurados e baseados na correta interpretação das respostas humanas, posto que diagnósticos precisos contribuem para prognósticos melhores^(30)^.

Os DEs com foco no problema representaram a segunda categoria mais utilizada, sendo “Dor aguda” e “Integridade da pele prejudicada” os mais elencados. Cabe destacar que alterações na integridade da pele estão comumente relacionadas a pacientes hospitalizados, em virtude dos procedimentos invasivos inerentes ao ambiente. Tal circunstância requer atenção, visto que procedimentos invasivos podem propiciar o desenvolvimento de infecções relacionadas à atenção à saúde, principalmente em pessoas que vivem com HIV, devido à imunossupressão^(22)^. Dados apontam que uma em cada quatro pessoas vivendo com HIV corre risco de ter o óbito como desfecho durante a hospitalização^(24)^.

Ampla revisão de literatura sobre os DEs em diferentes populações de pacientes e cenários de saúde apontou que “Dor aguda” foi o único DE identificado em todos os estudos analisados^(31)^, contrapondo os resultados da presente pesquisa, em que o referido DE foi elencado somente em 13 prontuários. As intervenções dos enfermeiros diante do manejo da dor tendem a priorizar a avaliação e o tratamento farmacológico, sugerindo a necessidade de treinamentos da equipe com ênfase no manejo não farmacológico da dor^(32)^. No contexto da atenção ao HIV, a dor crônica é reconhecida como uma comorbidade comum e uma ameaça à qualidade de vida dessas pessoas^(33)^.

Considera-se que fatores biológicos, psicológicos e sociais podem estar associados ao aumento da dor, como o estigma inerente ao HIV, déficit no apoio social, acesso reduzido aos serviços, atividade imunológica desregulada, inflamação e danos teciduais causados por coinfecções relacionadas à infecção^(33)^. Posto que a dor é uma experiência subjetiva e sua percepção considerada complexa e multidimensional, compreender essas influências é fundamental para implementação de estratégias personalizadas e eficazes para controle e manejo da dor^(34)^.

Identificou-se, ainda, que os DEs referentes à promoção da saúde e síndrome representaram a minoria da amostra, com somente um diagnóstico de cada tipo elencado, sendo, respectivamente, representados por “Disposição para enfrentamento melhorado” e “Síndrome do trauma de estupro”. Da mesma forma, entre os diagnósticos elencados para o cuidado de pacientes em Unidade de Terapia Intensiva, destacou-se a ausência de DE com foco na promoção da saúde, com predominância de diagnósticos de risco e com foco no problema^(7)^. Isso pode indicar que os enfermeiros estão focando a atenção somente nas complicações apresentadas, não considerando as possíveis formas de promoção da saúde, sendo esses DEs também relevantes no contexto de evitar/minimizar outros agravos à saúde.

O Brasil tem dedicado esforços à pesquisa sobre a acurácia dos DEs, reforçando a importância da qualidade dessa prática para a enfermagem brasileira^(35)^. Em síntese, DEs acurados contribuem para a elaboração de um plano de cuidados direcionado às pessoas que vivem com HIV, fomentando a organização gerencial e assistencial das ações dos enfermeiros, ao direcionar os profissionais para um conjunto de fenômenos para o qual o seu olhar precisa estar atento, considerando todo o contexto dessas pessoas^(35)^.

### Limitações do estudo

Por tratar-se de pesquisa com coleta de dados em prontuários, algumas informações pertinentes para reforçar os dados do estudo se encontravam ausentes e/ou não se tem certeza da veracidade; por esse motivo, não foram apresentadas neste manuscrito, como estado civil, orientação sexual, situação ocupacional e adesão à TARV. Ainda, a avaliação dos fatores de risco e características definidoras consideradas para cada DE poderia complementar a avaliação da acurácia.

Recomenda-se cautela na generalização dos resultados, visto que o estudo não contemplou uma análise estatística inferencial, o que pode ter limitado a profundidade dos achados. Ressalta-se, entretanto, que os resultados obtidos estão em consonância com a literatura.

### Contribuições para a área da enfermagem

O estudo promove contribuições para o avanço da enfermagem e da taxonomia NANDA-I, ao proporcionar evidências que podem auxiliar na qualidade da prática assistencial da enfermagem enquanto ciência no cenário nacional. Ressalta-se e reforça-se a importância de os profissionais enfermeiros se apropriarem da temática, uma vez que se constitui uma ferramenta norteadora do cuidado, ao dignificar a promoção do conforto, bem-estar e qualidade de vida durante o período de internação hospitalar, e, principalmente, na vida cotidiana das pessoas que vivem com HIV.

## CONCLUSÕES

Os DEs analisados foram, em sua maioria, acurados, indicando que os enfermeiros possuem um raciocínio clínico apurado, ao mesmo passo que a categoria de acurácia nula apresentou relevância na amostra, sugerindo que os conhecimentos acerca do tema precisam ser fortalecidos na prática profissional, sendo a importância de DEs assertivos reconhecida pelos gestores. Ressalta-se, ainda, que a temática deve ser incluída desde os cursos de graduação até os cursos de pós-graduação em enfermagem, estendendo-se para a prática profissional por meio da educação permanente em saúde, devido à sua relevância para a prática de enfermagem.

Os DEs prevalentes elencados pelos enfermeiros foram “Risco de infecção”, “Risco de integridade da pele prejudicada” e “Dor aguda”, tendo foco nos riscos e nos problemas, sugerindo que o cuidado de enfermagem também está direcionado nesse sentido. Isso fomenta a necessidade de uma atenção voltada à promoção da saúde, inclusive no âmbito hospitalar, considerando que, devido ao caráter crônico da infecção pelo HIV, será crucial que, após a alta, esses indivíduos sigam buscando por outros serviços que compõem a RAS.

As informações obtidas neste estudo podem, ainda, direcionar para o perfil da população que vive com HIV que está sendo hospitalizada devido ao agravamento da infecção, orientando pesquisadores e formuladores de políticas quanto às necessidades de saúde dessa população.

Para pesquisas futuras, sugere-se que os autores considerem estudos que contemplem as características e fatores de risco comumente apresentados por pessoas que vivem com HIV, buscando atender às demandas de saúde por meio de intervenções de enfermagem direcionadas e efetivas.

## Data Availability

Os dados da pesquisa estão disponíveis no corpo do artigo.
